# User perceptions of intelligent offloading diabetic footwear

**DOI:** 10.3389/fendo.2024.1380525

**Published:** 2024-08-07

**Authors:** Sarah L. Hemler, Carolyn M. Sommerich, Jorge C. Correia, Zoltan Pataky

**Affiliations:** ^1^ Faculty of Medicine, University of Geneva, Geneva, Switzerland; ^2^ Unit of Therapeutic Patient Education, WHO Collaborating Centre, Geneva University Hospitals, Geneva, Switzerland; ^3^ Department of Integrated Systems Engineering, The Ohio State University, Columbus, OH, United States; ^4^ Faculty Diabetes Centre, Faculty of Medicine, University of Geneva, Geneva, Switzerland

**Keywords:** adherence, diabetic foot, foot pressure, footwear, off-loading

## Abstract

**Aims:**

Adherence to therapeutic footwear is vital for effective diabetic foot ulcer prevention and treatment. Understanding the key adherence factors and potential barriers is important for footwear design and implementation. Our team is creating intelligent offloading footwear to prevent lower extremity amputations in people living with diabetes (PLwD). This exploratory study assessed the ability of the established Unified Theory of Acceptance and Use of Technology (UTAUT) model to predict behavioral intention to use or recommend this intelligent offloading footwear by PLwD, caregivers of PLwD, or medical professionals treating PLwD.

**Methods:**

Online and paper questionnaires were implemented to assess the impact of the UTAUT model factors (performance expectancy, effort expectancy, social influence, facilitating conditions) and psychosocial factors (attitude, anxiety, self-efficacy) on the overall behavioral intention to use the footwear. Furthermore, factors influencing potential acceptance and rejection of the footwear were explored.

**Results:**

Patients (4.0/5) and medical professionals (4.1/5) showed a behavioral intention to “agree” to use or recommend the footwear when it becomes available. Structural equation modeling showed that the UTAUT constructed model may not be the best indicator for behavioral intention here based on a lack of statistical significance. However, the logistic regression modeling showed that the social influence for PLwD (p=0.004) and the attitude toward the footwear for medical professionals (p=0.001) may be the most important when designing and implementing the footwear, though several other factors (performance expectancy, effort expectancy, facilitating conditions, and self-efficacy) were also important for one or both of these populations. Additionally, cost and clinician support were shown to be important factors influencing potential acceptance of the footwear.

**Conclusions:**

The study found promising intention to use the intelligent footwear in the future. This highlights the need to continue future development and implementation of the footwear to incorporate these results, thus improving the likelihood of high adherence of the footwear.

## 1 Introduction

One in ten adults globally has diabetes (1). Diabetic foot ulcers affect 19-34% of people living with diabetes (PLwD) and are a leading cause of amputations in this population (2–5). Diabetes-related lower-extremity amputations lead to an increase in illness-related costs and a diminished quality of life compared to the general population (6). Studies have shown an increase in the prevalence of psychiatric disorders among this group of PLwD including depression and posttraumatic stress disorder (7). Lower-extremity amputations are also related to significant early and long-term post-operative mortality (8). There is a need to contribute to the currently available preventive modalities for these foot ulcers that can lead to amputations.

Diabetic foot ulcers are primarily due to peripheral neuropathy, or a lack of sensation in the extremities, coupled with high pressures under the foot (9). When the high pressures are not perceived by the patient and therefore not offloaded during walking, they can lead to callus formation, subcutaneous hemorrhaging, and then a foot ulcer which, if infected, can lead to an amputation. There are many ways to prevent and treat foot ulcers including surgical approaches (10, 11); however, the most prevalent approaches are pressure offloading interventions. Current methods include non-removable (e.g., total contact casts) and removable (e.g., removable cast walkers, custom insoles and footwear) interventions, with the former possibly increasing pre-existing challenges such as mobility and daily living activity impairments, low quality of life, and stigmatization (12–16). Removable interventions are often preferred by PLwD for convenience and adaptability to lifestyle (17); they have had varied success, but research has shown that using the interventions has positive effects (18, 19).

For removable interventions, one of the main limiting factors for ulcer healing is non-adherence. Higher adherence is associated with ulcer healing (20) and thus a lower risk for amputation. However, fewer than 50% of PLwD and neuropathy, who are especially at risk for ulceration, wear their therapeutic footwear (newly-prescribed, custom-made) for more than 60% of daytime hours (21, 22). Other studies have shown that 28-60% of patients adhere to footwear recommendations (23).

Among some of the most influential factors of footwear dissatisfaction and thus adherence in this population are footwear weight (21, 24), comfort (21), style (25), and the perceived opinions of others (26). Furthermore, Maslow’s Hierarchy of Needs, as described for product development research, maintains that these factors must be accounted for, especially to satisfy the need categories of social needs (e.g. belonging), esteem needs (e.g. positive self-evaluation and dignity) and self-actualization needs (e.g. self-fulfillment and beauty) (27, 28). Research has also shown that higher adherence is linked to the patient having paid employment, a current or previous foot ulcer, and an understanding of the risks associated with neuropathy along with the conviction that the footwear aids ulcer healing (29). In terms of the practical use of the footwear, important factors for adherence include the satisfaction level of the follow-up for the footwear, self-efficacy, storage location/type of footwear at home, and consistency of the type of footwear worn (29). Although these aesthetic needs may be considered by some to be less important than physiological needs for people with diabetes at risk for ulceration, they still greatly influence acceptability. Furthermore, it is essential to appeal to the patient themselves and address misconceptions regarding foot health, types of footwear, and footwear fit through therapeutic patient education to ensure that the overall foot care and footwear are appropriate (30–32). To address and design for all the needs of an individual, user-centered design principles such as user feedback assessments should be implemented during the design process.

Assessments of technology acceptance and planned behavior provide effective methods for determining current usage and potential adherence (33). For products in any stage of design/development, from ideation to finishing, technology acceptance models may be especially useful for gauging *usefulness* and *desirability*, two topics that bridge the engineering, marketing, and design teams during the design process (27). A review of technology acceptance models has shown that a combination of previous methods may provide a more modern approach that is applicable in many fields (34–36); the Unified Theory of Acceptance and Use of Technology (UTAUT) pulls from eight other user acceptance models (35). Venkatesh, et al. (36) simplified the UTAUT model from 32 main effects and four moderators to one with four primary constructs that showed to be direct determinants of intention and usage behavior (35). In this previous study, these four determinants are defined: *1*) performance expectancy (PE) as the perceived usefulness of the technology, *2*) effort expectancy (EE) as the perceived ease of use of the technology, *3*) social influence (SI) as the degree to which clinicians and caregivers support the use of the technology, and *4*) facilitating conditions (FC) as the degree to which the user believes they possess the cognitive and physical ability to use the technology (35). These four constructs and the three associated psychosocial moderators of attitude (ATT), anxiety (ANX), and self-efficacy (SE) together have been used to estimate the perceived acceptance of healthcare-related technology along with potential barriers (34, 35, 37).

In this exploratory study, we present the UTAUT model with moderating psychosocial factors applied to intelligent offloading footwear (IOF) that is under development by our team at the University of Geneva, Geneva University Hospitals, and École Polytechnique Fédérale de Lausanne (38). The IOF is designed as an everyday shoe for PLwD and neuropathy who are at risk for developing ulcers; the footwear is designed to sense the location of high plantar pressures and then actively adjust the contour of the insole to reduce these high plantar pressures. The purpose of this exploratory study was to investigate the predictors from the UTAUT model and psychosocial factors analysis of behavioral intention to use the IOF by PLwD, caregivers, and medical professionals.

## 2 Materials and methods

### 2.1 Study summary

In this cross-sectional survey design study, a paper-and-pencil or an online questionnaire was given to PLwD (DM), caregivers of PLwD (CG), and medical professionals who care for PLwD (MP). The questionnaire employed general information and footwear preferences along with 26 questions to understand the predictive capability of the UTAUT model and moderating psychosocial factors to predict behavioral intention to use the IOF. The 26 questions relating to the UTAUT model were based on previous work (35, 37) and the other questions were designed for general understanding of design implications. The English language version was written by the first author (SH) – a native English speaker – based on previous work (35, 37). This version was translated into French by a native French speaker who also speaks English fluently and who was familiar with the work, but does not have topic-relevant expertise. The French and English versions were then compared by several other native French speakers who are fluent in English and have topic-relevant expertise (i.e., researchers with experience in movement and perception), in consultation with the first author (SH) until consensus was reached that the two versions were comparable. Furthermore, the French paper version was distributed to the patients prior to the distribution of the online questionnaire; this allowed for the investigating researchers to adjust the questionnaire according to any common questions which arose concerning the language. As there were no concerns for the translation after the paper version, the French version remained the same for the online version.

Hypothesis 1 (*H1*): The UTAUT model (PE, EE, SI, FC) predicts behavioral intention (BI) by DM to use and MP to prescribe the intelligent footwear when it becomes available.

Hypothesis 2 (*H2*): Attitude (ATT), anxiety (ANX), and self-efficacy (SE) moderate the impact of UTAUT on the behavioral intention prediction.

### 2.2 Questionnaire format

#### 2.2.1 Pencil-and-paper version

PLwD in the Geneva University Hospitals (Department of Endocrinology, Diabetes, Nutrition and Therapeutic Patient Education) were recruited for a questionnaire to understand their perceptions of the proposed IOF (39). Questionnaires were conducted from June to August 2022. People with diabetes who had active or past foot ulcers and who were cognitively able to fill out a questionnaire in English or French were included in the study. Reasons for exclusion of data included those who did not complete the questionnaire. Ethical approval was granted by the University Commission for Ethical Research in Geneva for the paper and online versions (CUREG 2022-03-35).

#### 2.2.2 Online version

A questionnaire similar to the paper questionnaire was made available online (LimeSurvey GmbH, Hamburg, Germany) from October 2022-January 2023 to DM, CG, and MP populations. As in the paper questionnaire, versions were made available in English and French.

#### 2.2.3 Questionnaire sections

The paper and online questionnaires consisted of similar questions separated into 5 sections: *1*) General description of the footwear, *2*) person-specific information, *3*) diabetes-specific questions, *4*) UTAUT model and psychosocial moderators, *5*) footwear design.

Section 1 stated that our team is developing IOF for people with diabetes with the intent to reduce the likelihood of ulcers and amputations. A brief description was given concerning how the footwear would sense the location and magnitude of high pressures under the foot and then actively adjust the insole to redistribute the high pressures to reduce the risk of ulceration. The respondents were then instructed that during the 15-20 minute questionnaire, they should imagine that they or the person for whom they care/oversee would 1) receive the footwear from a medical professional, 2) wear the shoes while walking around each day, 3) remove the shoes and plug them into the charger after each day of wear, and 4) unplug the shoes from the charger and put them on the next day.

Section 2 gathered demographical and other user-specific data (sex, age, country of residence, level of education) while Section 3 asked users to fill out diabetes-specific information (numbers of years since diagnosis, type of diagnosis, presence of a previous foot ulcer, active and shoe-wearing hours per day) for themselves (DM) or for the person for whom they are a caregiver (CG).

Section 4 consisted of questions regarding the UTAUT model and psychosocial factor (PE, EE, SI, FC, ATT, ANX, SE, and BI). These questions were adapted to the role of the person answering the questions. For example, a question such as “I would find the footwear useful for managing my foot health” was tailored such that “my” would be replaced with either “my care recipient’s” (for CG) or “my patient’s” for MP. For each of these questions, users were asked to rate their agreement with the question on a 5-point Likert scale (1=strongly disagree, 2=disagree, 3=neutral, 4=agree, 5=strongly agree; verbal descriptors were present at each number). Questions for ANX have the opposite connotation compared to the other categories; a smaller number denotes less anxiety toward using the footwear. Behavioral intention (BI) was measured by two questions which asked the participants to rate how likely they would be to wear or recommend the shoes when they became available (**Table 1**).

**Table 1:** Participant characteristics by questionnaire type, language, gender, age, region, and education.

Descriptive Statistics						
*Characteristic*	DM		CG		MP	
	n	*%*	n	*%*	n	*%*
**Total Sample**	48	*37.5*	11	*8.6*	69	*53.9*
Questionnaire Type						
Online	25	*19.5*	11	*8.6*	69	*53.9*
Paper	23	*18.0*	0	*0*	0	*0*
Language						
English	20	*15.6*	7	*5.5*	50	*39.1*
French	28	*21.9*	4	*3.1*	19	*14.8*
Sex						
Female	17	*13.3*	6	*4.7*	25	*19.5*
Male	29	*22.7*	4	*3.1*	42	32.8*
No Response	2	*1.6*	1	*0.8*	2	*1.6*
Age						
19-30	4	*3.1*	1	*0.8*	11	*8.6*
31-40	0	*0*	2	*1.6*	16	*12.5*
41-50	8	*6.3*	1	*0.8*	20	*15.6*
51-60	13	*10.2*	2	*1.6*	13	*10.2*
61-70	12	*9.4*	2	*1.6*	8	*6.3*
70+	11	*8.6*	2	*1.6*	1	*0.8*
No Response	0	*0*	1	*0.8*	0	*0*
Region						
Africa	0	*0*	1	*0.8*	3	*2.3*
Asia	1	*0.8*	1	*0.8*	9	*7.0*
Australia	3	*2.3*	1	*0.8*	3	*2.3*
Europe	35	*27.3*	5	*3.9*	38	*29.7*
North America	8	*6.3*	2	*1.6*	14	*10.9*
South America	1	*0.8*	0	*0*	2	*1.6*
No Response	0	*0*	1	*0.8*	0	*0*
Education (years)						
1-5	0	*0*	0	*0.0*	2	*1.6*
6-8	0	*0*	0	*0.0*	2	*1.6*
9-12	5	*3.9*	0	*0.0*	1	*0.8*
13-16 (e.g., Bachelors degree)	17	*13.3*	5	*3.9*	12	*9.4*
>16 (e.g., Masters/ Doctoral degree)	11	*8.6*	6	*4.7*	52	*40.6*
No Response/undetermined*	2/13*	*1.6/15.4*	0	*0*	0	*0*

Sample frequency is expressed as % of all participants, N = 128.

*Inconsistency in question format left 13 paper questionnaires that would've been between 1 and 12 years as undetermined.

Section 5 consisted of multiple-choice questions concerning why the person with diabetes might not want to use the footwear, what might change their mind in accepting the footwear, and preferences for footwear closure type, cost, and outer design.

### 2.3 Data analysis

**Figure 1:**
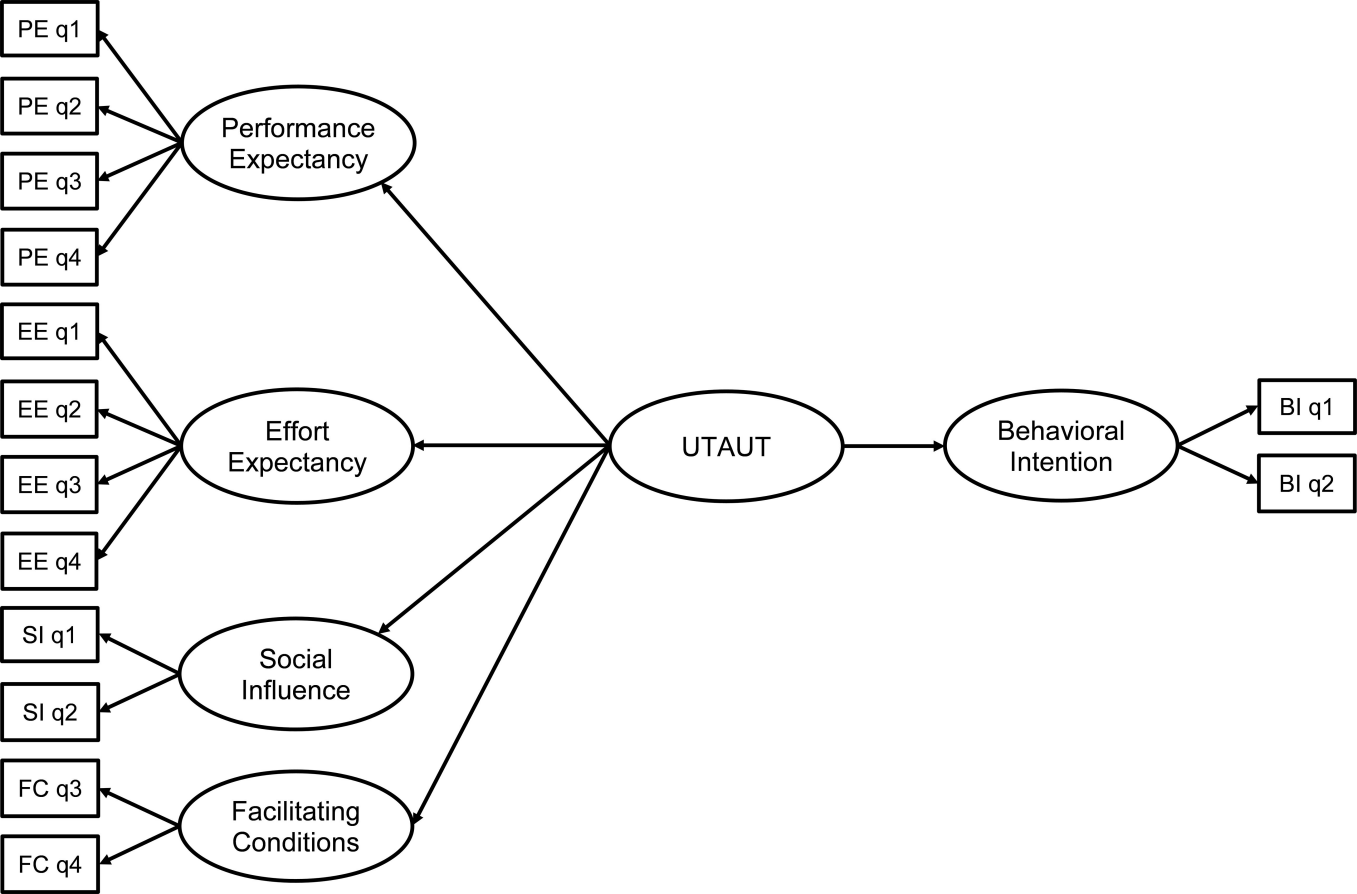
SEM analysis structure for *H1* where each question (q#) was incorporated into the model.

The data were anonymized and statistical analyses were performed by the researchers including descriptive statistics (mean and SD), frequency counts, and percentages of total participants within each role. Further analyses were separated by role due to the nature of the question responses being perception-based. Therefore, for each group, Cronbach’s alpha measure of construct reliability with a threshold set at 0.7 or higher for acceptable reliability (40) was used to measure the reliability of the measures within the UTAUT model and within the general psychosocial factors. Multiple questions in each factor were accounted for by predicting a latent variable for each factor. Structural equation modeling (SEM) was used based on the work of Kohnke, et al. (35) to assess *H1* (**Figure 1**). If the results for the SEM for H1 showed agreement, then linear regression analysis would be used for *H2*.

Factor loadings (FL) >0.7, Comparative Fit Index (CFI) ≥ 0.95, Root Mean Square Error of Approximation (RMSEA) ≤ 0.06, and the ratio of chi-square to the degrees of freedom (χ2/df) < 2 were used to evaluate the SEM (41–47). IBM SPSS (28.0.1.1 (14)) and AMOS (v29.0.0, SPSS) were used for all tests and Stata/SE 17.0 was used to calculate the predicted latent variables.

## 3 Results

### 3.1 Primary results

A total of 23 people (DM) completed the paper questionnaire and 105 people (DM, CG, and MP) completed the online questionnaire in French or English for a total of 128 responses (**Table 1**). For the paper questionnaire, there were 45 out of the 68 possible patients who did not complete the questionnaire or did not participate due to disinterest, language barriers, cognitive difficulties, or bilateral amputations that rendered footwear not useful. For the online questionnaire, as there was a low number of CG, this population was only included for descriptive statistics as shown in **Table 1**.

In total, the DM population was generally older than the MP population and there were generally more males than females in the DM and MP groups. The most responses for each population came from Europe and secondly from North America. The majority of respondents in all populations possessed at least a bachelor’s degree.

The mean value for all question groups in the UTAUT model was above 4.0 (agree) except for the facilitating conditions for the MP populations (**Table 2**). Within the UTAUT factors, the scores for the DM group were generally higher than the MP group scores. Within the psychosocial factors, the general attitude was positive (>4.0) and the anxiety level was nearly neutral for DM and MP groups. The self-efficacy scores were less than 4.0 for both groups. Cronbach’s alpha was above the threshold of 0.7 for all categories except for facilitating conditions and self-efficacy in the DM group.

**Table 2:** Construct reliability and validity of the UTAUT model with moderating variables.

Characteristic	DM				MP			
	Mean^++^	SD	α^+++^	FL	Mean	SD	α	FL
UTAUT Model (PE, EE, SI, FC)			0.926	0.78			0.938	0.56
Performance Expectancy (4 items)	4.2	0.8	0.885	0.84	4.2	1.0	0.900	0.78
- I would find the footwear useful for managing my foot health	4.1	1.0		0.52	4.3	1.0		0.63
- Using the footwear would enable me to take better care of my feet	4.2	0.8		0.94	4.3	1.0		0.91
- Using the footwear would allow me to be more involved and productive in my foot care	4.3	0.8		0.87	4.1	1.1		0.77
- If I use the footwear, I believe I would reduce my chance of developing a foot ulcer	4.3	0.7		0.80	4.1	1.0		0.81
Effort Expectancy (4 items)	4.3	0.8	0.832	0.86	4.1	1.0	0.914	0.89
- I expect my interaction with the footwear will be clear and understandable	4.3	0.7		0.74	4.0	1.0		0.89
- I expect that the footwear will be easy for me to use	4.3	0.9		0.70	4.1	1.0		0.93
- I expect learning to use the charging system will be easy for me	4.3	0.8		0.83	4.0	1.1		0.84
- I expect to become skilled at using, or it will be easy for me to use the footwear as part of my daily routine	4.4	0.7		0.69	4.2	0.9		0.75
Social Influence (2 items)	4.2	0.8	0.872	0.90	4.2	0.8	0.867	0.92
- People who influence my behavior (clinicians & caregivers) would support me using (or would think that I should use) this footwear	4.2	0.9		0.88	4.2	0.8		0.84
- People who are important to me (family, friends, and colleagues) would support me using (or would think that I should use this footwear)	4.2	0.8		0.88	4.2	0.7		0.72
Facilitating Conditions (2 items)	4.3	0.7	**0.512**	1.20	3.8	1.0	0.783	0.93
- I believe I would have the competence necessary to use this footwear	4.5	0.6		**0.55**	3.8	1.0		0.89
- This footwear would be better than other methods I use to manage my foot health	4.1	0.7		**0.64**	3.7	1.0		0.72
Moderating Variables (ATT,ANX,SE)								
Attitude (4 items)	4.3	0.9	0.797	--	4.4	0.8	0.867	--
- Using this footwear would be a good idea	4.4	0.7			4.4	0.7		
- Using this footwear would make managing my foot health more interesting	4.3	0.8			4.2	0.8		
- I would like to wear this footwear	4.3	0.9			4.4	0.7		
- I would be willing to wear this footwear in a clinical trial for at least 2 months	4.2	1.2			4.4	0.8		
Anxiety (5 items) *	2.5	1.2	0.852	--	3.2	1.2	0.794	--
- I would feel anxious using the footwear	2.2	1.1			2.5	1.3		
- I worry that I might not use the footwear appropriately	2.4	1.2			3.3	1.1		
- I would be concerned that I might forget to charge the batteries	2.5	1.3			3.7	1.0		
- I would be concerned that I might not like the style of the footwear	3.2	1.2			3.4	1.0		
- The footwear would be somewhat intimidating to me	2.0	1.0			3.0	1.1		
Self-Efficacy (3 items)	4.0	1.1	**0.308**	--	3.8	0.8	0.850	--
- I could use the footwear and complete the daily charging of the battery if there was no one around to assist me	4.1	1.1			3.6	0.8		
- I could put on the footwear and use them by myself	4.4	0.7			3.8	0.8		
- I could use the footwear and complete the daily charging of the battery if I could call someone for help if I got stuck or confused	3.4	1.3			4.0	0.7		
Dependent Variable (BI)								
Behavioral Intention (BI)	4.0	0.83	0.916	--	4.1	0.8	0.781	--
- I think I would use the intelligent footwear in my daily life when it is available	4.0	0.79		0.91	4.0	0.7		0.85
- I will plan to use the intelligent footwear in my daily life when it is available	4.0	0.87		0.93	4.1	0.8		0.76

^++^Mean based on response option scale: 1=strongly  disagree, 3=neutral, 5=strongly agree.

^+++^α refers to Cronbach’s alpha.

*Lower values indicate less anxiety.

Bold values indicate that the value outside of the acceptable range.

In the SEM model, the factor loadings for the UTAUT model, as a whole, were 0.8 for the DM group and 0.56 for the MP group. The CFI and RMSEA were 0.863 and 0.134 for the DM group and 0.881 and 0.130 for the MP group. The ratio of chi-square to the degrees of freedom (χ2/df) was 1.862 for the DM group and 2.144 for the MP group.

### 3.2 Exploratory analysis

The results of the parameters as shown in Section 2.3 – Data Analysis showed that the UTAUT may not be an effective model to examine the relationships among the variables. To explore these relationships, the following exploratory analysis was conducted similar to previous work (48–50).

#### 3.2.1 Exploratory methods

Previously established methods of using logistic regression modeling were modeled to explore the relationships between all categories and the behavioral intention to use the footwear (48–50).

Step 1: The predicted latent variable for each UTAUT model factor and psychosocial factor was determined. In each population, a univariate logistic regression model was tested for each factor in relation to the behavioral intention (BI) to use the footwear. BI was converted into a binary factor with the average of the Likert values less than 4 indicated as negative responses to BI and values of 4 and above were indicated as positive responses to BI. A predicted variable could not be created for the FC factor in the MP group due to a lack of responses for the two questions. Therefore, univariate logistic regression was conducted for the remaining predicted factors for a total of 13 models tested (2 populations * 7 factors [4 UTAUT and 3 psychosocial]). Any factor that was not determined to be statistically significant with a Wald test p-value >0.20 was excluded from further analysis. Multicollinearity between the statistically significant factors in each population was tested by performing linear regression analyses including the BI as the dependent variable and the significant factors in each group as the independent factors (in all possible combinations).

Step 2: The factors that proved significant in Step 1 were incorporated into a backward selection multivariate logistic regression to select important factors to keep (p-value < 0.10). A categorical backward selection was not used as there were too few variables to conduct this analysis as in previous work (48, 50).

#### 3.2.2 Exploratory results

In the DM and MP groups respectively, there were 33/48 and 53/69 responses indicating a positive intention to use the IOF when considering BI as a binary variable (**Table 3**). Univariate logistic regression analysis showed that for the DM group, PE, EE, SI, FC, and ATT were significant (p<0.20) and that for the MP group, PE, EE, ATT, and SE were significant. Both groups showed no multicollinearity among the significant factors (DM: VIF [1.13-2.57], Tolerance [0.39-0.89], r [.385-.468]; MP: VIF [1.09-2.25], Tolerance [0.45-0.92], r [.265-.496]) (51). Therefore, each of these factors was carried over to the multivariate logistic regression for the respective group (**Table 4**). Backward selection showed that the multivariate regressions were eventually reduced to having only one factor (univariate regressions) with the significance level set to *p* < 0.10 (Wald test). SI (OR=3.5, *p*=0.004) was the only remaining factor in the DM group and ATT (OR=30.9, *p*=0.001) was the remaining factor in the MP group.

**Table 3:** Univariate logistic regression analysis.

Univariate Logistic Regression Analysis^a^						
	BI (<4)^b^	BI (4-5)	OR	95% CI		p
DM						
PE	15	33	3.8	1.2	11.8	0.022
EE			7.6	1.3	44.5	0.024
SI			3.5	1.5	8.1	0.004
FC			2.9	1.0	8.1	0.045
ATT			7.3	1.2	44.4	0.031
MP						
PE	16	53	1.9	1.0	3.5	0.054
EE			1.7	0.9	3.2	0.086
ATT			30.9	3.8	250.4	0.001
SE			4.2	1.4	12.0	0.008

a significant factors (p < 0.20).

b BI <4 signifies a lack of intention to use.

**Table 4:** Multivariate logistic regression analysis.

Multivariate Logistic Regression Analysis - backward selection^c^							
		BI (<4)^b^	BI (4-5)	OR	95% CI		p
**DM**	SI	15	33	3.5	1.5	8.1	0.004
**MP**	ATT	16	53	30.9	3.8	250.4	0.001

^c^significant factors (p < 0.10).

^b^BI <4 signifies a lack of intention to use.

The most predominant concern by all roles for why PLwD may not wear the footwear was described as the cost (**Table 5**). However, the main reason stated by DMs and MPs as to why a patient may change their mind to accept the footwear was also if the cost of the footwear was covered by insurance. The amount of money that someone would be willing to spend annually on footwear was recorded. For the paper questionnaire, the conversion rate from CHF to USD was based on the start date of the first survey. As there was a maximum of 5% fluctuation in the exchange rate in this period, the risk of error was small. For the online questionnaire, an error prevented the collection of currency type for the responses. Therefore, the currency was defaulted to the country location of the respondent and was limited to the following countries: USA [USD], Canada [CAD], European countries using the Euro [EUR], Switzerland [CHF], United Kingdom [GBP], and Australia [AUS]. Other currencies and amounts were excluded from the analysis. Currency was converted to USD for the online questionnaires according to the conversion rate of the date the questionnaire was received. Across the paper and online questionnaires, the average cost (in USD) that PLwD may be willing to pay annually for the footwear was estimated to be $322 for DMs, $158 for CGs and $448 for MPs.

**Table 5:** Reported multiple choice questions – reasons for potential rejection or acceptance of the footwear and preferred style.

Reasons to not wear the footwear	DM			CG			MP		
	n	% of all n	% of responses	n	% of all n	% of responses	n	% of all n	% of responses
Reasons to not wear the footwear									
Not interested	1	2	4	1	9	10	6	9	5
Already a good way to manage foot health	0	0	0	1	9	10	5	7	4
Don't think it would help current situation	4	8	16	0	0	0	1	1	1
Worry that the footwear would be too expensive	9	19	36	6	**55**	**60**	50	**72**	***41***
Too fearful to try the footwear in case they don't work well with feet	1	2	4	0	0	0	21	30	17
Too much work to charge the batteries	2	4	8	1	9	10	17	25	14
Not applicable - would wear the footwear	8	17	32	1	9	10	22	32	18
Reasons that might change someone's mind to accept the footwear									
Nothing	4	8	11	1	9	7	1	1	0
Clinician insists/encourages to wear/accept them	3	6	8	6	**55**	40	36	**52**	18
A family member encourages to wear/accept them	1	2	3	0	0	0	33	***48***	16
Clinician/caregivers encourages use due to another hospital admission	1	2	3	1	9	7	32	***46***	16
Full cost covered by insurance	10	21	28	4	36	27	53	**77**	26
Not having to charge the footwear batteries so often	2	4	6	2	18	13	32	46	16
Not applicable - would wear the footwear	15	31	***42***	1	9	7	18	26	9
Preferred footwear closure type									
Normal laces	7	15	21	4	36	33	15	22	14
Elastic laces	6	13	18	2	18	17	22	32	21
Hook & loop fastener (e.g., Velcro)	10	21	29	4	36	33	46	**67**	***44***
Zipper	4	8	12	2	18	17	14	20	13
Not applicable - the closure type doesn't matter	7	15	21	0	0	0	7	10	7

Percentages above 50% are bold and highlighted, and those above 40% are in bold and italics.

The potential cost of the footwear was the largest perceived prohibitor to acquisition and subsequent use of the shoes. Otherwise, the general, open-ended questions as to why people would not wear the footwear fell into 2 main categories: aesthetics and usability. The main concerns for people with diabetes were the style (aesthetics) and that the footwear would not fit their feet or that the battery in the shoe could be dangerous (usability). For the MPs, the main concerns were centered around being able to effectively encourage use of the footwear and durability (usability), along with the importance of the style for PLwD (aesthetics).

The aesthetic preferences of the participants were varied. In general, the preferred type of closure was stronger for medical professionals as a hook and loop fastener (Velcro^®^). In the questionnaire, participants were shown two images of potential aesthetic designs: one sport and one formal. The sport design was preferred by most of the DMs (20/24 respondents), while the preferences of the MPs were split between the formal (29/65 respondents) and the sport (36/65 respondents).

## 4 Discussion

The high potential usage scores based on the UTAUT model suggest that there could be high adoption of the new IOF when it becomes available. Based on established thresholds, the Structural Equation Modeling showed that a UTAUT model may not be the most indicative of behavioral intention for PLwD nor with medical professionals. However, the supplementary, exploratory analysis showed that for PLwD, all the UTAUT model factors (PE, EE, SI, FC) may individually positively influence Behavioral Intention (BI) with Social Influence (of caregivers, medical professionals, and family members) being the most influential factor in predicting BI (based on multivariate backward selection). For the Medical Professional (MP) group, univariate analysis showed the significance of Performance Expectancy and Effort Expectancy, from the UTAUT model, as well as Attitude and Self-Efficacy in predicting BI. In the MP group, the Attitude toward the shoes was the prominent factor for predicting BI in the multivariate analysis. Apart from the UTAUT model, cost/affordability of the footwear was also identified by participants as an important factor that could influence adoption of the footwear.

The results from this study align with previous findings. In the current study, for PLwD, the impact of external opinions and the advice of medical professionals (Social Influence) was shown to be an important factor for wearing this footwear (multivariate backward selection regression). This is consistent with previous work that showed that stigmatization and confirmation from MPs that recommended footwear is helpful and important for adherence (26, 29). Furthermore, for MPs in the current study, the Attitude toward the footwear proved to be the most influential factor for them recommending the footwear to their PLwD. This result is consistent with previous results that showed that there must be buy-in and a positive attitude of a device from an MP before recommending the device to a patient (52–55). The cost was shown to be an influential part of the potential wear of the shoes which is consistent with previous work indicating that this topic is important for understanding potential adherence (56).

Certain limitations to this research should be stated as well. Our intelligent footwear was presented as a concept and as such was only described and not shown in physical form to the participants. This may have limited responses, though it may have also avoided certain biases. For those who were invited to complete the questionnaire online, there was no option for completing a paper copy of the questionnaire. As such, some potential participants who did not have internet access or were uncomfortable using technology may not have participated. This could be an important missing group, given that the shoes being developed are “high tech”. Therefore, the study pool may have been limited for this reason and because of the missed patients in the paper questionnaire study. The study population does not represent global opinions, because most of the participants were located in Europe or North America. A larger sample size could also aid in the use of the UTAUT which was limited due to the number of participants. Furthermore, in this study, Cronbach’s alpha assumptions were not met for the FC and SE questions in the DM group which was primarily due to the small number of questions. Therefore, this set of questions may not be the most influential for future design considerations. Due to limited resources, forward translation followed by a sort of expert committee and pilot testing were conducted as mentioned in the methods for the translation from English to French. With a larger target audience and more available resources, a rigid structure for the translation validation could be performed in the future (57).

Future designs of the questionnaire may incorporate more comprehensive imagery or use description of the footwear to provide respondents with a more realistic understanding of the footwear and in turn that will allow respondents to provide a more accurate perception of the footwear. Implementation of the footwear should include a focus on ensuring that there is a positive attitude instilled in potential end-users (persons with diabetes) and those who care for them (Medical Professionals), to affect the overall attitude toward the footwear and the social influence parameter. Furthermore, attention to shoe affordability will address an important potential barrier for many prospective end users.

Overall, this study showed that the UTAUT model, as a whole, and three previously identified psychosocial factors did not necessarily predict stakeholder behavioral intention, but rather specific factors in the model predicted the intention to use or recommend the footwear for people with diabetes and medical professionals, respectively. In attracting future patients with diabetes and their medical professionals to use new footwear, this work suggests that information given should focus on targeting a positive social influence and attitude toward the footwear. The work is useful not only for the design of the presented IOF, but also for future intelligent footwear that may emerge with advances in technology.

## Data availability statement

The raw data supporting the conclusions of this article will be made available by the authors, without undue reservation.

## Ethics statement

The studies involving humans were approved by University Commission for Ethical Research in Geneva. The studies were conducted in accordance with the local legislation and institutional requirements. The participants provided their written informed consent to participate in this study.

## Author contributions

SH: Conceptualization, Data curation, Formal analysis, Investigation, Methodology, Project administration, Visualization, Writing – original draft, Writing – review & editing. CS: Conceptualization, Formal analysis, Methodology, Validation, Visualization, Writing – review & editing. JC: Investigation, Writing – review & editing. ZP: Conceptualization, Funding acquisition, Methodology, Project administration, Supervision, Writing – review & editing.
